# Penicillin–streptomycin influences macrophage mechanical properties and microenvironment mechano-sensation^1^^1^

**DOI:** 10.1016/j.mbm.2025.100173

**Published:** 2025-12-31

**Authors:** Shiqi Hu, Buwei Hu, Jing Yang, Rui Liu, Yang Song, Yufan Zheng

**Affiliations:** a Institute of Biomedical Engineering, West China School of Basic Medical Sciences & Forensic Medicine, Sichuan University, Chengdu, 610041, China; b Department of Bioengineering, University of California, Los Angeles, CA90095, USA; c Department of Materials Science, Fudan University, Shanghai, 200433, China

**Keywords:** Macrophage, Penicillin–streptomycin (pen-strep), Cell stiffness, Cell adhesion, Mechanotransduction

## Abstract

Penicillin–streptomycin (pen-strep) is routinely included in cell culture media, yet its impact on macrophage mechanics has not been systematically examined. Here, we show that pen-strep treatment increases macrophage stiffness in a time-dependent manner, while adhesion strength is only transiently affected. Morphological analysis revealed that pen-strep promotes cell spreading on PDMS rubber, collagen I, laminin, poly-amino acids, and poly-RGD peptides, but reduces spreading on type IV collagen, indicating altered extracellular matrix sensing in a context-dependent fashion. Gene expression assays further demonstrated upregulation of YAP-1 and TAZ and downregulation of β1 integrin, consistent with reprogramming of mechanotransduction pathways. Consequently, pen-strep elevated intracellular ROS, suppressed the M1 gene spectrum, induced heterogeneous M2-associated responses, and impaired phagocytic capacity. Collectively, these findings identify pen-strep as a modulator of macrophage stiffness, ECM mechano-sensation, polarization, and key immune functions, raising concerns about its routine use in mechanobiology research and clinical applications.

## Graphical abstract

**Figure:**
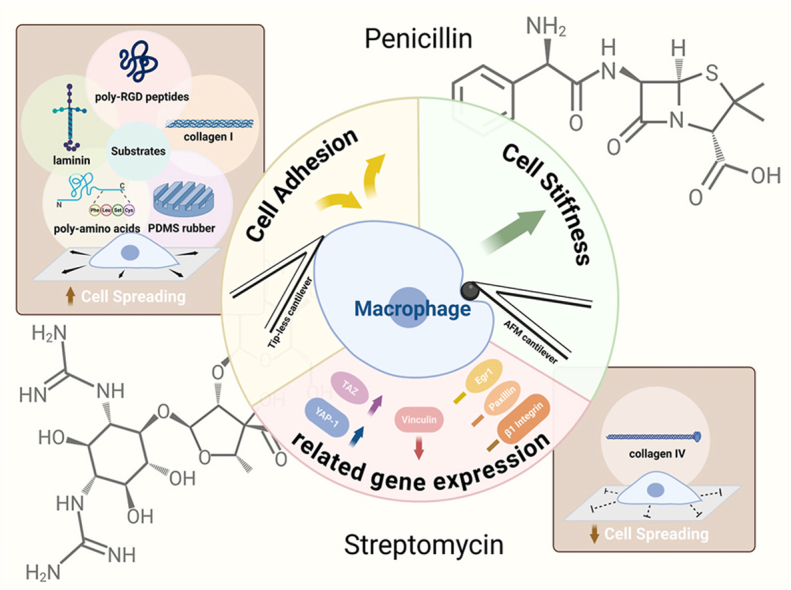

## 1 Introduction

Macrophages, originated from hematopoietic stem cell–derived monocytes, are integral members of the mononuclear phagocyte system. As core components of innate immunity, macrophages act as first responders against pathogens and serve as a crucial bridge linking innate and adaptive immune responses. They perform diverse functions, including immune surveillance, phagocytosis, antigen presentation, and immune regulation, while exhibiting functional plasticity and tissue specificity 1, 2, 3. Macrophages have been classified into two polarized states: M1 macrophages are pro-inflammatory, producing cytokines such as TNF-α, IL-1β, IL-6, IL-12, and IL-23, thereby amplifying antimicrobial and antitumor immune responses; in contrast, M2 macrophages are associated with anti-inflammatory and tissue-repair functions, by secreting IL-10 and TGF-β to suppress excessive inflammation while remodeling the extracellular matrix to promote tissue regeneration.^4^

Studies have demonstrated that chemical and physical cues within the microenvironment can modulate the mechanical properties of macrophages.^5^^,^^6^ The mechanical properties of cells reflect the function and state of cells to a certain extent. Specifically, macrophage cellular stiffness indicates macrophage phenotypic transition and functional status.^7^^,^^8^ M1 macrophages generally exhibit higher stiffness compared with M2 macrophages.^8^ By stimulating with lipopolysaccharide (LPS), activated macrophages show enhanced cellular stiffness and adhesive capacity relative to their resting counterpart.^8^^,^^9^ Furthermore, alterations in macrophage cellular stiffness have been shown to influence key immune functions, including phagocytosis, migration, cytokine secretion, and reactive oxygen species (ROS) production.^10^ Although studies have extensively explored how common chemicals and cytokines (LPS, IFN-γ, IL-4/IL-13, GM-CSF, M-CSF^11^^,^^12^) induce changes in macrophage mechanics, the effects of commonly used pharmacological agents on macrophage cellular stiffness remain largely unexplored and warrant further investigation.

*In vitro* cell culture technology, as one of the cornerstones of modern life sciences, has greatly promoted and reshaped the appearance of basic scientific research by providing a simplified yet controllable system that enables investigations not feasible *in vivo*. And penicillin and streptomycin (pen-strep), as broad-spectrum antibiotics, were routinely included in cell culture media. However, in addition to antimicrobial function, the potential impact of pen-strep on the physiology and function of macrophages has remained largely unexplored.^13^^,^^14^

In this study, we demonstrated that pen-strep treatment upregulates cellular stiffness, reprograms microenvironmental sensation, and mechano-phenotypic transition of macrophages. In detail, after pen-strep treatment, macrophage cellular stiffness increased while cell adhesion was only transiently affected, meanwhile, cell morphology responded differently to ECM mechanics and distinct ECM components. The gene expression analysis indicated that *Taz*, *Egr-1*, and Yap-1 transcription were enhanced, while the expression of integrin β1 decreased, which might be able to explain the mechano-phenotypic switching, as the downregulation of β1 integrin may influence cell attachment to different ECMs. Functionally, pen-strep elevated intracellular ROS, broadly repressed the M1 genes, induced a heterogeneous response on M2-associated genes, and strongly impaired phagocytic capacity. Taken together, these findings highlight the need to consider the effects of pen-strep in *in vitro* macrophage studies to ensure the reliability of experimental outcomes. Moreover, the broader impact of pen-strep on other cell types warrants further systematic investigation.

## 2 Materials and methods

### 2.1 Cell culture

RAW264.7 cells were obtained from the American Type Culture Collection (ATCC, Manassas, VA) and maintained in Advanced Dulbecco's Modified Eagle's Medium (DMEM; Invitrogen) supplemented with 10 % fetal bovine serum (FBS; Fisher Scientific, Houston, TX). Cells were seeded onto 24-well plates, cover glasses (12 mm; Fisher Scientific, 12CIR-1.5) or PDMS rubber coated with 10 mg/mL ECM or peptide (poly-RGD peptide, collagen I, collagen IV, poly-amino acid or laminin), and used at ∼80 % confluence. Cultures were maintained at 37 °C incubator supplied with 5 % CO_2_, washed with phosphate-buffered saline (PBS; Corning, 46-013-CM), and passaged using a medium-flush method. For some experiments, cells were treated with the commercial penicillin-streptomycin antibiotic mixture at a final concentration of 1 % (v/v), which corresponds to the standard working concentration used in routine mammalian cell culture (approximately 100 U/mL penicillin and 100 μg/mL streptomycin), for 24 h.

### 2.2 Reverse transcription quantitative polymerase chain reaction (RT-qPCR)

RAW264.7 cells were cultured with small disks (diameter 3 cm, thickness 0.5 cm) of different material groups in 6-well plates. Cells were maintained in DMEM supplemented with 10 % FBS under standard conditions (37 °C, 5 % CO_2_). The expression of cell-attachment- and mechanotransduction-related genes, including *Tafazzin*, *Egr1*, *Yap1*, *Vcl*, *Pxn*, *Itgb1*, *Tnf*, *iNos*, *Il1b*, *Cxcl9*, Arg*1*, *Mrc1*, *Il10*, *Ccl7,* and *Gapdh*, was analyzed at defined time points, with the first measurement performed 1 day after culture. RNA was extracted using a total RNA kit (Omega Bio-Tek, Norcross, GA, USA). Complementary DNA was synthesized (at 37 °C for 15 min and 85 °C for 5s) using a PrimeScript RT reagent kit (Takara, Shiga, Japan). qRT-PCR assay was performed using the SYBR premix Ex Taq reagent (Takara) with a CFX Connect Real-Time PCR Detection System (Bio-Rad, Hercules, CA, USA). Primers were designed and synthesized by Sangon Biotech Co. Ltd. (Shanghai, People's Republic of China) using Primer Premier software (PREMIER Biosoft, Palo Alto, CA, USA). The primer sequences are presented in Table S1. Relative gene expression was calculated using the 2^−ΔΔCT^ method, normalized to glyceraldehyde-3-phosphate dehydrogenase (GAPDH) as the internal control.

### 2.3 Atomic force microscope (AFM) and single-cell force spectroscopy (SCFS)

**Fig. 1:**
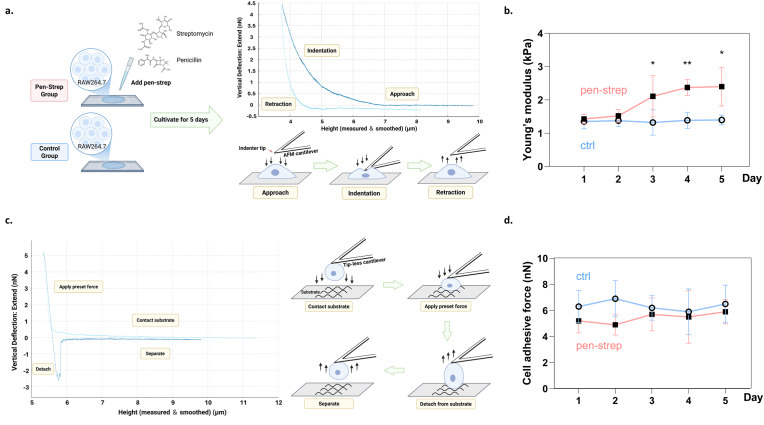
**Pen-strep increase macrophage stiffness but not adhesive strength.** (a) Schematic illustration of AFM measurement of cell stiffness. (b) Time-dependent changes in cellular stiffness of macrophages w/o pen-strep treatment, n = 30, Two-way ANOVA with Tukey's multiple comparisons test. (c) Schematic illustration of SCFS measurement of cell adhesion force. (d) Time-dependent changes in adhesion force of macrophages w/o pen-strep treatment, n = 30, Two-way ANOVA with Tukey's multiple comparisons test. Schematics in (a) and (c) were created with BioRender. In this study, error bars represent s.e.m., ns: no significance, ∗: p < 0.05, ∗∗: p < 0.01, ∗∗∗: p < 0.001, ∗∗∗∗: p < 0.0001.

Cell stiffness was measured using an optical lever AFM (Nanoscope V, Digital Instruments) with a tipless “V”-shaped cantilever (NP-O10, Bruker, spring constant: 0.06 N/m), as shown in Fig. 1,^15^ mounted on a micromanipulator (Narishige). All mechanical measurements were performed within a temperature-controlled environmental chamber (Bruker, BioHeater) maintained at 37.0 °C, with additional temperature regulation provided by a Tokai Hit thermo-controlled stage to ensure physiological conditions.

The indentation measurements were performed on the central apical region of the cell. Force–distance curves were collected at contact times of 0, 5, and 10 s under a maximum load of 5 nN. Young's modulus was calculated from indentation curves using Sneddon's modification of the Hertz model. The indentation depth was kept <500 nm to avoid damage.

Here, a tipless cantilever was used, the indenter geometry was approximated as a flat-ended punch. The loading force *F* was related to the indentation depth *δ* according to:

$$F=2E^{*}a\delta$$

where *E∗* is the reduced elastic modulus and a represents the effective contact radius of the indenter. Assuming cells behave as an incompressible material with a Poisson's ratio of *ν* = 0.5, the apparent Young's modulus (*E*) was calculated as:

$$E^{*}=\frac{E}{1-v^{2}}$$

For single-cell force spectroscopy, individual RAW264.7 cells were attached to a ConA-functionalized tipless cantilever. The cantilevers were sequentially coated with biotin-BSA, streptavidin, and biotin-ConA to ensure strong and specific binding to cell-surface glycoconjugates. During attachment, the cantilever was gently pressed onto a single cell with a contact force of 1 nN for 2 s, followed by a 10-minute stabilization period to establish firm adhesion and prevent detachment. Attachment quality was verified under an inverted microscope, and cells showing lateral movement were excluded. The cell-bearing cantilever was then used to detach the cell from substrates in PBS (pH 6.0) under the same loading conditions. Both the approach and retraction (pull-off) speeds were set to 5 μm/s to minimize hydrodynamic drag and ensure reproducible force–distance curves. The SCFS detachment experiments were also conducted under the same temperature-controlled conditions (37 °C). Adhesion forces were quantified from force–distance curves analyzed with Nanoscope Analysis software (Bruker). Each condition was tested with freshly prepared probes, and three measurements at random positions were averaged. This AFM–SCFS approach enabled quantitative assessment of macrophage stiffness and adhesion after pen-strep treatment.

### 2.4 F-actin staining

Cells were fixed with pre-chilled 4 % paraformaldehyde (PFA) for 20 min at room temperature, then samples were permeabilized with 0.5 % Triton X-100 (Sigma, T8787) in PBS for 20 min. After that, samples were blocked with 1 % Normal Serum and Gamma Globulins (NDS) in PBS for 1 h at room temperature. After that, the F-actin was stained with Alexafluor 488-conjugated phalloidin (1:400) for 1 h, and the nuclei were counterstained with 4′,6-Diamidino-2-Phenylindole (DAPI, IntrogenTM Molecular ProbesTM, D3571) for 5 min following the manufacturer's instructions. Between these steps, the slides were washed with PBS three times for 5 min to remove unbonded molecules. A Zeiss microscope was used to image macrophage morphology. ImageJ) was used to measure the roundness of each macrophage. Cell morphology was quantified in terms of “roundness” using ImageJ (NIH). Roundness was calculated based on the best-fit ellipse for each cell according to the formula:

$$Roundness=\frac{4\cdot Ar\mathrm{e}a}{\pi \cdot {MajorAxis}^{2}}$$

where Area is the projected cell area and Major Axis is the length of the major axis of the ellipse. A value of 1 indicates a perfect circle, while lower values denote increased elongation. For each experimental condition, a minimum of 50 cells from three independent experiments were analyzed.

### 2.5 Intracellular reactive oxygen species (ROS) measurement

Intracellular reactive oxygen species (ROS) levels were measured using a DCFH-DA-based fluorescent probe according to the manufacturer's instructions (Reactive Oxygen Species Assay Kit, Beyotime Biotechnology, China). Briefly, RAW264.7 cells were seeded in 6-well plates and subjected to the indicated treatments. After treatment, the culture medium was removed, and the cells were gently washed once with PBS. DCFH-DA was diluted in serum-free DMEM at a ratio of 1:1000 to obtain a final concentration of 10 μM.

The staining solution was added to the cells (500 μL per well), followed by incubation at 37 °C in a humidified atmosphere containing 5 % CO_2_ for 20 min. After incubation, cells were washed three times with serum-free DMEM to remove excess probe that had not entered the cells. Fluorescence signals were immediately observed and captured using an inverted fluorescence microscope under identical exposure settings. For each experimental group, 100 cells were randomly selected for quantitative analysis. Intracellular ROS levels were quantified based on the fluorescence intensity of dichlorofluorescein (DCF) using ImageJ software (NIH).

### 2.6 Immunofluorescence staining

Cell proliferation was assessed by immunofluorescent staining of Ki67. RAW264.7 cells were cultured on glass coverslips under the indicated experimental conditions. After treatment, cells were washed with PBS and fixed with 4 % paraformaldehyde for 10 min at room temperature. Fixed cells were permeabilized with 0.5 % Triton X-100 in PBS for 10 min and blocked with 1 % Bovine Serum Albumin (BSA) in PBS for 1 h at room temperature.

Cells were then incubated overnight at 4 °C with an anti-Ki67 primary antibody (HuaBio, China, HA721115) at a dilution of 1:500. After washing three times with PBS, samples were incubated with the appropriate fluorophore-conjugated secondary antibody for 1 h at room temperature in the dark. Nuclei were counterstained with DAPI for 5 min. Coverslips were mounted with antifade mounting medium and imaged using a fluorescence microscope. Ki67-positive cells were quantified as a percentage of total DAPI-positive nuclei from randomly selected fields. For each group, 15 random fields were selected for analysis, and the percentage of Ki67-positive cells was calculated relative to the total number of DAPI-positive nuclei.

### 2.7 Macrophage phagocytosis assay

Macrophage phagocytic activity was evaluated using pHrodo™ Red BioParticles (Thermo Fisher Scientific, P35361), which emit fluorescence in acidic phagosomes. RAW264.7 cells were seeded in 96-well plates at a density of 20,000 cells per well and allowed to adhere for at least 1 h before treatment with the indicated compounds for 2 h pHrodo BioParticles were resuspended in PBS to prepare a 1 mg/mL stock solution and briefly vortexed. The suspension was diluted 1:10 in complete culture medium and added to the cells. Cells were incubated at 37 °C for 30 min to 3 h (typically 2 h) to allow phagocytosis. After incubation, cells were washed with PBS to remove non-internalized particles and fixed with 4 % paraformaldehyde for 10 min at room temperature. Nuclei were counterstained with DAPI (1:1000) for 5 min. Fluorescent images were acquired using a fluorescence microscope under identical imaging conditions. For each condition, at least 200 cells were randomly selected, and the percentage of phagocytic cells was calculated based on the presence of intracellular red fluorescence.

### 2.8 Transwell migration assay

Cell migratory capacity was evaluated using a Transwell assay. Briefly, Transwell inserts with a porous membrane (pore size: 8 μm) were placed into 24-well plates. RAW264.7 cells were suspended in low-serum (1 %) DMEM and seeded into the upper chamber at a defined density. The lower chamber was filled with low-serum (1 %) DMEM supplemented with 20 ng/mL M-CSF as a chemoattractant.

Cells were incubated at 37 °C in a humidified atmosphere with 5 % CO_2_ for a 24-h duration to allow migration. After incubation, non-migrated cells on the upper surface of the membrane were gently removed using a cotton swab. Migrated cells on the lower surface were fixed with 4 % paraformaldehyde and stained with 0.1 % (w/v) crystal violet. Images were captured using an inverted microscope, and the number of migrated cells was quantified from multiple random fields using ImageJ software. For each experimental group, migrated cells were quantified from five randomly selected microscopic fields using ImageJ software.

## 3 Results

### 3.1 Pen-strep increase macrophage stiffness but not adhesive strength

Macrophage cellular stiffness is closely associated with its functional state and cytokine secretion capacity.^8^ Using AFM, cell stiffness can be measured and calculated based on the force-displacement curve of the interaction between the cantilever and the sample (Fig. 1a). To explore whether macrophage cellular stiffness was shifted by pen-strep, macrophages were cultured on glass slides and treated with pen-strep for five days, during which cellular stiffness was measured daily. The results showed that under pen-strep treatment, the stiffness of macrophages increased significantly after 24 h and reached the peak on day 5 at around 2.5 kPa (Fig. 1b). Notably, a pronounced rise in cell stiffness was observed between day 2 and day 3, with the elastic modulus increasing from 1.5 to 2.3 kPa within 24 h. Except for cellular stiffness, macrophage adhesion strength is another indicator of macrophage phenotype and function.^8^ Similar to AFM, the single-cell force spectroscopy (SCFS) measures cell-ECM adhesive force based on the force applied on the cell to detach from the substrate (Fig. 1c). It is important to note that here the aim of the SCFS measurements was to assess the overall cellular adhesion capacity under the defined conditions, instead of identifying specific receptor-mediated (e.g., integrin) interactions, which typically require longer contact times to develop. Accordingly, our assay primarily captures early-stage, largely non-specific adhesion events. The results revealed that the adhesion capacity of macrophages decreased slightly after two days, reaching the lowest point at around 5 nN, but recovered to the baseline level (∼6 nN) by day 3 and remained stable thereafter (Fig. 1d).

### 3.2 Pen-strep influence macrophage microenvironment sensation

**Fig. 2:**
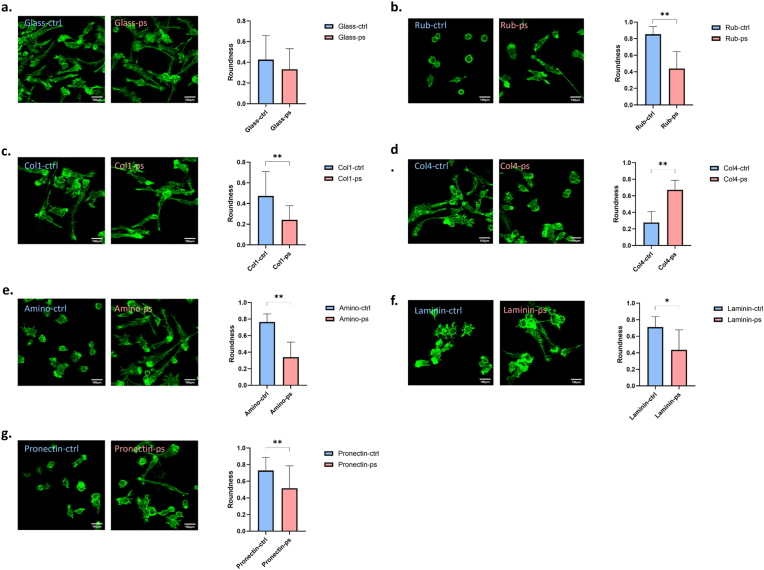
**Pen-strep influence macrophage microenvironment sensation.** Cell morphology quantified by roundness after 24 h culture on (a) glass, (b) PDMS rubber and (c) collagen I-/(d) collagen IV-/(e) poly-amino acid-/(f) laminin-/(g) poly-RGD peptide-coated surfaces w/o pen-strep treatment, n = 50, two-tailed Student's *t*-test.

Based on the observed changes in cell stiffness after treatment, we hypothesized that pen-strep treatment may influence macrophage morphology and their ability to sense the microenvironment. Since both ECM mechanics and ECM ligand–mediated biochemical signaling govern microenvironmental mechanosensing, macrophages were treated w/o pen-strep, seeded on different substrates or ECM-coated surfaces, and cultured for 24 h, followed by phalloidin staining to visualize and analyze cell morphology. After 24 h of treatment, the roundness of macrophages on glass slides showed no significant difference compared with the control group (Fig. 2a). In contrast, although macrophages typically exhibit impaired adhesion on PDMS rubber, pen-strep treatment compensated for this effect and promoted cell spreading (Fig. 2b). Beyond substrate sensing, we further examined whether pen-strep modulates macrophage interactions with ECM ligands. Type I, IV collagen, and laminin are common ECM proteins found in tissues. We found pen-strep reduced cell roundness on type I collagen- and laminin-coated surfaces (Fig. 2c–f), whereas it increased roundness on type IV collagen (Fig. 2d). In addition to ECM proteins, how macrophages respond to polypeptides that mimic ECM functions were also tested. Poly-amino acids and poly-RGD peptides are widely used to facilitate cell attachment.^16^ Pen-strep treatment was also found to promote cell spread on these polypeptides-functionalized surface (Fig. 2e–g). Collectively, these results suggest that pen-strep alters macrophage sensing of microenvironmental cues, including both substrate mechanics and ECM ligand composition.

### 3.3 Pen-strep effect stiffness- and adhesion-related gene expression

**Fig. 3:**
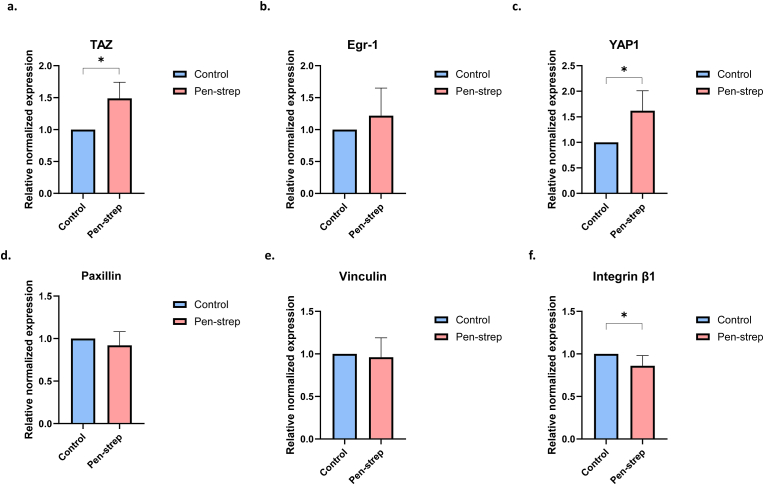
**Pen-strep effect stiffness- and adhesion-related gene expression.** Relative mRNA expression level of (a) *Taz*, (b) *Egr-1*, (c) *Yap-1*, (d) *paxillin*, (e) *vinculin*, and (f) *β1 integrin* w/o pen-strep treatment for 24h, n = 3, two-tailed Student's *t*-test.

To investigate the mechanism of these phenomena, we analyzed the expression of transcription factors and membrane proteins associated with cellular stiffness and cell adhesion. After treating macrophages on glass with pen-strep for 24 h, mRNA transcription level of TAZ, Egr-1, YAP-1, vinculin, paxillin, and β1 Integrin was evaluated by RT-qPCR. The results indicated pen-strep treatment upregulated the expression of TAZ and YAP-1 (Fig. 3a–c). Furthermore, pen-strep downregulates β1 integrin, a critical molecular “clutch^17^” in ECM mechanosensing and focal adhesion maturation process (Fig. 3f). In contrast, the expression levels of paxillin, vinculin, and Egr-1 remained largely unchanged following treatment.

### 3.4 Pen-strep–induced mechanical alterations are associated with changes in macrophage phenotype and function

**Fig. 4:**
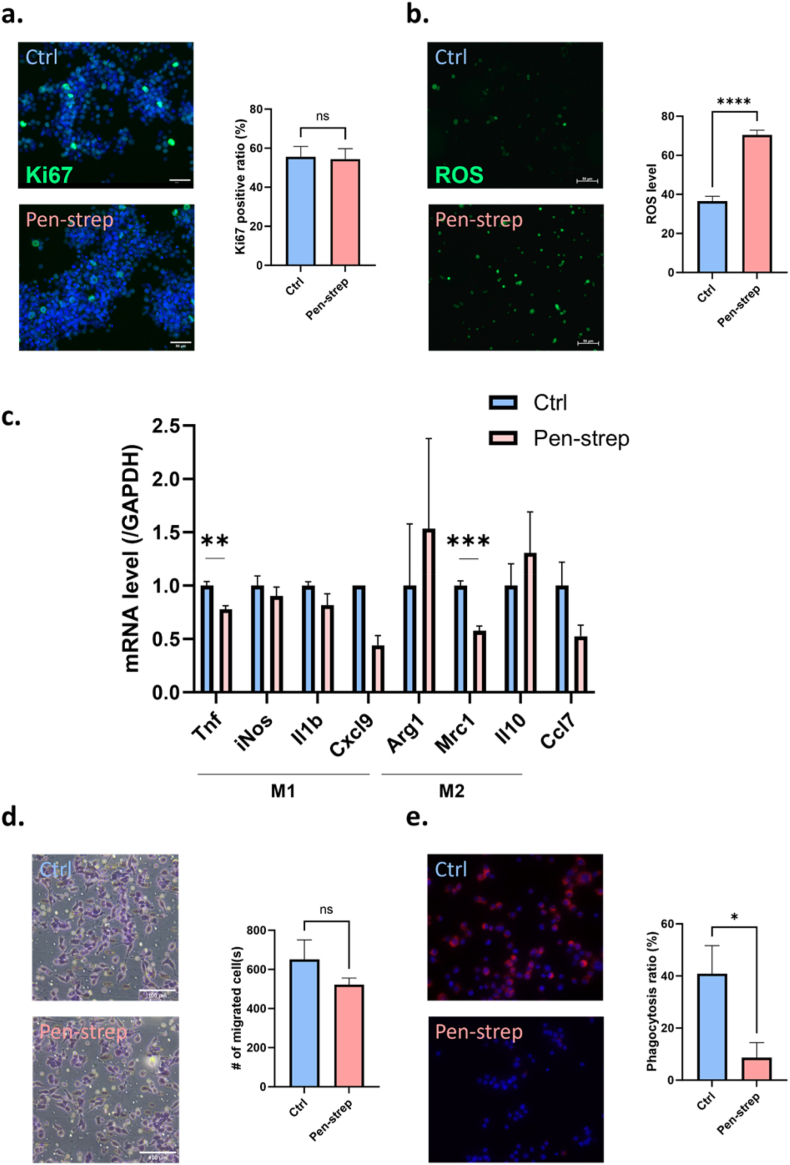
**Pen-strep–induced mechanical alterations are associated with changes in macrophage phenotype and function.** (a) Representative immunofluorescence images of Ki67 staining in macrophages cultured w/o pen-strep treatment for 24 h, n = 15, two-tailed Student's *t*-test. (b) Representative fluorescence images of intracellular reactive oxygen species (ROS) levels in macrophages with or without pen-strep treatment, and corresponding quantitative analysis, n = 100, two-tailed Student's *t*-test. (c) Relative mRNA expression level (normalized to *Gapdh*) of M1/2 polarization-associated marker and cytokines w/o pen-strep for 24 h, n = 3, multiple *t*-test with Welch's correction. (d) Representative phase-contrast images of macrophages subjected to 8-μm transwell assay w/o pen-strep treatment for 24 h, n = 5, two-tailed Student's *t*-test. (e) Representative images of phagocytosis assay w/o pen-strep treatment for 2 h, with quantification of phagocytosis ratio, n = 200, two-tailed Student's *t*-test.

Given that macrophage stiffness is closely linked to both phenotypic state and functional behavior, we examined whether pen-strep–induced mechanical alterations were accompanied by changes in macrophage phenotype. We first assessed parameters reflecting the cellular general status. Ki67 immunofluorescence staining showed no significant difference in the proportion of proliferating cells between control and pen-strep–treated macrophages (Fig. 4a), indicating that pen-strep does not measurably affect macrophage proliferation despite the observed upregulation of the mRNA level of YAP/TAZ. In contrast, intracellular reactive oxygen species (ROS) levels were significantly increased following pen-strep treatment (Fig. 4b), suggesting enhanced oxidative stress. We next investigated whether these changes were accompanied by shifts in macrophage polarization. Interestingly, pen-strep treatment significantly downregulated pro-inflammatory M1 marker spectrums and pro-inflammatory cytokines and chemokines, including Tnf and *Cxcl9*, whereas *iNos* and *Il1b* only tended to decrease. Markers associated with M2-like or regulatory phenotypes exhibited a heterogeneous response: Arg*1* and the anti-inflammatory cytokine *Il10* were significantly upregulated, while *Mrc1* (*CD206*) was markedly downregulated (Fig. 4c). Additionally, Ccl7, a cytokine independent of macrophage polarization, also tended to decrease, collectively suggesting a non-canonical phenotypic state characterized by selective modulation of cytokine and chemokine expression. Finally, we assessed whether macrophage cellular functions are affected. Migration assays revealed a modest reduction in the number of migrated cells following pen-strep treatment (Fig. 4d). Moreover, phagocytic capacity was significantly reduced in pen-strep–treated macrophages compared with controls (Fig. 4e). Given that both directional migration and phagocytosis critically depend on actomyosin dynamics and cell mechanical properties, these findings support the conclusion that pen-strep–induced mechanical alterations translate into selective functional remodeling rather than global functional suppression.

## 4 Discussion

In the present study, our data indicate that pen-strep can mediate macrophage mechanical properties, morphology, ECM mechano-sensation, corresponding gene expression and several key functions. Thus, pen-strep becomes a chemical that can influence macrophage stiffness and gene expression patterns. Thus, the usage of pen-strep in cell culture media for macrophage mechanobiology study becomes a concern. Also, results imply that pen-strep used in antibiotic treatment may influence macrophage function in the inflammation process.

Under pen-strep stimulation, the cell stiffness of macrophages cultured on glass increased. Previous studies have suggested that cell stiffness is closely associated with cell spreading.^18^ Specifically, as macrophage stiffness increases, cell roundness typically decreases, consistent with reports showing that cells with larger spreading areas exhibit higher stiffness. During spreading, cell height decreases while nuclear stiffness increases, ultimately contributing to an overall rise in cellular stiffness.^19^ However, in our experiments, macrophage roundness on glass did not decrease significantly under pen-strep treatment (Fig. 2a). This discrepancy suggests that transcriptional regulation may contribute to the observed changes in cell mechanics. Indeed, further experiments revealed that pen-strep treatment enhanced the expression of YAP-1 and TAZ, key transcriptional regulators of cell stiffness and macrophage phenotypic transition.^20^ YAP-1 and TAZ modulate cellular mechanics through the Hippo pathway, where their nuclear accumulation promotes cytoskeletal remodeling and increased stiffness.^21^ Thus, the upregulation and nuclear localization of YAP-1 and TAZ under pen-strep treatment are likely to activate Hippo signaling, thereby mediating the observed increase in macrophage stiffness.

In contrast to the increase in cell stiffness, pen-strep treatment transiently impaired macrophage adhesion capacity on day 2, but adhesion strength recovered to baseline levels after three days. Typically, cell adhesion is regulated by integrin-mediated focal adhesion maturation, which depends on integrin clustering and phosphorylation of focal adhesion–associated tyrosine kinases.^22^ Integrins can also recruit Src kinase and talin to activate downstream pathways that regulate adhesion. Within this process, vinculin stabilizes focal adhesions by directly interacting with talin and actin,^23^ while paxillin, once phosphorylated by Src, associates with actin to further modulate cell–ECM attachment.^24^ However, after one day of pen-strep treatment, the expression of both paxillin and vinculin remained largely unchanged. Instead, pen-strep specifically downregulated β1 integrin, a key mediator of cell–ECM interactions. These results suggest that the transient reduction of β1 integrin on the cell surface may account for the impaired adhesion observed on day 2. Since paxillin- and vinculin-dependent pathways were not significantly affected, adhesion strength subsequently recovered by day 3. The precise signaling mechanisms underlying this transient effect of pen-strep remain to be elucidated.

Cell morphology is strongly influenced by microenvironmental mechanosensing, which is governed by both substrate mechanics and ECM ligand–mediated biochemical signaling.^25^^,^^26^ β1 integrin interacts with various ECM components and plays a central role in regulating cell morphology, for instance, cell spreading is promoted when integrins engage with fibronectin and laminin.^22^^,^^27^ Interestingly, although β1 integrin expression was reduced under pen-strep treatment, macrophages still exhibited spreading on type I collagen, laminin, poly-RGD peptide, poly-amino acid–coated substrate, but shrank on type IV collagen–coated substrate following pen-strep exposure. These findings suggest that, beyond β1 integrin, pen-strep may activate alternative signaling pathways to regulate cell morphology and ECM recognition. Since different combinations of integrin α and β subunits confer distinct affinities for ECM ligands.^28^ It is possible that other integrin subunits are upregulated to compensate for the reduction of β1 integrin, thereby maintaining or even enhancing ECM binding. Further investigation is required to elucidate the precise mechanisms by which pen-strep modulates macrophage–ECM interactions.

Functionally, pen-strep did not alter proliferation but markedly elevated intracellular ROS and reshaped cytokine expression. The mixed transcriptional profile (downregulation of certain M1 markers alongside upregulation of Arg1 and IL-10) points to a non-canonical immune state. These findings reinforce the link between oxidative stress, cytoskeletal remodeling, and altered mechanical properties. In addition, pen-strep reduced phagocytic capacity and tended to impair the migration capacity, which could be explained by increased stiffness-induced compromised cellular, especially cytoskeletal flexibility.

Taken together, our findings demonstrate that pen-strep not only alters macrophage mechanics and adhesion but also reprograms their functional state by elevating oxidative stress, reshaping cytokine expression, and impairing migration and phagocytosis. These effects are likely mediated through mechano-sensation–related pathways, including YAP/TAZ and integrin-β1. Such multifaceted influences highlight that pen-strep is an active modulator of macrophage mechanobiology. Consequently, its potential confounding effects on cellular mechanics, phenotype, and function should be carefully considered in studies of immune response, phagocytosis, and mechanotransduction.

## CRediT authorship contribution statement

**Shiqi Hu:** Writing – original draft, Visualization, Investigation, Data curation. **Buwei Hu:** Methodology, Investigation, Data curation. **Jing Yang:** Writing – original draft, Visualization. **Rui Liu:** Writing – original draft, Visualization. **Yang Song:** Writing – review & editing, Supervision, Resources, Project administration, Methodology, Conceptualization. **Yufan Zheng:** Writing – review & editing, Writing – original draft, Supervision, Project administration, Investigation, Conceptualization.

## Ethical approval

This study does not contain any studies with human or animal subjects performed by any of the authors.

## Declaration of competing interest

The authors declare that they have no known competing financial interests or personal relationships that could have appeared to influence the work reported in this paper.

## Data Availability

The data are available from the corresponding author on reasonable request.

## Appendix A Supplementary data

Multimedia component 1 Table S1. **Primer sequences.** The forward and reverse primer sequences (5′–3′) for *Tafazzin*, *Egr1*, *Yap1*, *Vcl*, *Pxn*, *Itgb1*, *Tnf*, *iNos*, *Il1b*, *Cxcl9*, Arg*1*, *Mrc1*, *Il10*, *Ccl7* and *Gapdh* genes used in the qPCR assays.
