# Supplementary material for: Penicillin–streptomycin influences macrophage mechanical properties and microenvironment mechano-sensation
Source: Mechanobiol Med. 2025 Dec 31;4(1):100173. doi: 10.1016/j.mbm.2025.100173 (PMC12816849; doi:10.1016/j.mbm.2025.100173)
Supplement: Multimedia component 1 — Table S1. Primer sequences. The forward and reverse primer sequences (5′–3′) for Tafazzin, Egr1, Yap1, Vcl, Pxn, Itgb1, Tnf, iNos, Il1b, Cxcl9, Arg1, Mrc1, Il10, Ccl7 and Gapdh genes used in the qPCR assays. [file mmc1.pptx]

## Slide 1
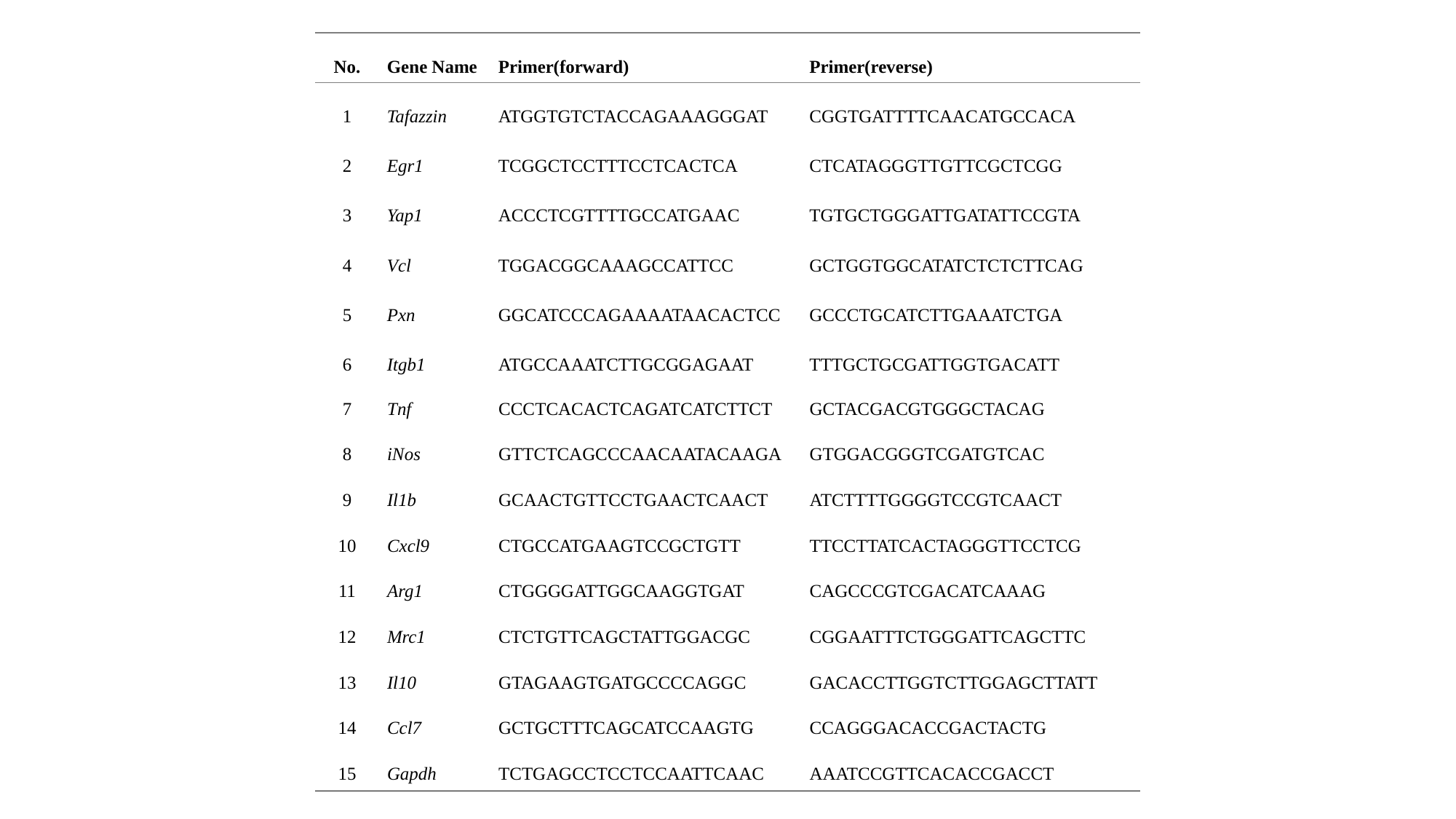

| No. | Gene Name | Primer(forward) | Primer(reverse) |
| --- | --- | --- | --- |
| 1 | Tafazzin | ATGGTGTCTACCAGAAAGGGAT | CGGTGATTTTCAACATGCCACA |
| 2 | Egr1 | TCGGCTCCTTTCCTCACTCA | CTCATAGGGTTGTTCGCTCGG |
| 3 | Yap1 | ACCCTCGTTTTGCCATGAAC | TGTGCTGGGATTGATATTCCGTA |
| 4 | Vcl | TGGACGGCAAAGCCATTCC | GCTGGTGGCATATCTCTCTTCAG |
| 5 | Pxn | GGCATCCCAGAAAATAACACTCC | GCCCTGCATCTTGAAATCTGA |
| 6 | Itgb1 | ATGCCAAATCTTGCGGAGAAT | TTTGCTGCGATTGGTGACATT |
| 7 | Tnf | CCCTCACACTCAGATCATCTTCT | GCTACGACGTGGGCTACAG |
| 8 | iNos | GTTCTCAGCCCAACAATACAAGA | GTGGACGGGTCGATGTCAC |
| 9 | Il1b | GCAACTGTTCCTGAACTCAACT | ATCTTTTGGGGTCCGTCAACT |
| 10 | Cxcl9 | CTGCCATGAAGTCCGCTGTT | TTCCTTATCACTAGGGTTCCTCG |
| 11 | Arg1 | CTGGGGATTGGCAAGGTGAT | CAGCCCGTCGACATCAAAG |
| 12 | Mrc1 | CTCTGTTCAGCTATTGGACGC | CGGAATTTCTGGGATTCAGCTTC |
| 13 | Il10 | GTAGAAGTGATGCCCCAGGC | GACACCTTGGTCTTGGAGCTTATT |
| 14 | Ccl7 | GCTGCTTTCAGCATCCAAGTG | CCAGGGACACCGACTACTG |
| 15 | Gapdh | TCTGAGCCTCCTCCAATTCAAC | AAATCCGTTCACACCGACCT |
